# Imaging for acute pelvic pain in pregnancy

**DOI:** 10.1007/s13244-014-0314-8

**Published:** 2014-02-18

**Authors:** Gabriele Masselli, Roberto Brunelli, Riccardo Monti, Marianna Guida, Francesca Laghi, Emanuele Casciani, Elisabetta Polettini, Gianfranco Gualdi

**Affiliations:** 1 Umberto I Hospital, Radiology Department, Sapienza University, Viale del Policlinico 155, 00161 Rome, Italy; 2 Department of Obstetrics and Gynecology, Sapienza University, Viale del Policlinico 155, 00161 Rome, Italy

**Keywords:** Acute pelvic pain, Pregnancy, Guidelines, Ultrasound, Magnetic resonance

## Abstract

Acute pelvic pain in pregnancy presents diagnostic and therapeutic challenges. Standard imaging techniques need to be adapted to reduce harm to the foetus from X-rays because of their teratogenic and carcinogenic potential. Ultrasound remains the primary imaging investigation of the pregnant abdomen. Magnetic resonance imaging (MRI) has been shown to be useful in the diagnosis of gynaecological and obstetric problems during pregnancy and in the setting of acute abdomen during pregnancy. MRI overcomes some of the limitations of ultrasound, mainly the size of the gravid uterus. MRI poses theoretical risks to the foetus and care must be taken to minimise these with the avoidance of contrast agents.

*Teaching Points*

• *Ultrasound and MRI are the preferred investigations for acute pelvic pain during pregnancy.*

• *Ultrasound remains the primary imaging investigation because of availability and portability.*

• *MRI helps differentiate causes of acute pelvic pain when ultrasound is inconclusive.*

## Introduction

A wide variety of diseases may appear with pain during pregnancy. The causes of pelvic pain in pregnancy can be classified in gynaecological causes and non-gynaecological causes.

Diagnosis of pelvic pain in pregnant women is confounded by several factors found in a normal pregnancy, such as nonspecific leukocytosis, displacement of abdominal and pelvic structures from their normal locations by the gravid uterus, a difficult abdominal examination, and nonspecific nausea and vomiting [1–3].

Therefore a prompt and accurate diagnosis and treatment are essential for the well-being of the mother and the foetus, and imaging is commonly requested to clarify the clinical picture and expedite diagnosis.

Given the established risks to the foetus from radiation exposure, ultrasound (US) and magnetic resonance imaging (MRI) are the preferred imaging investigations [4–6].

US is a rapid, safe and readily available imaging modality that does not require the administration of intravenous contrast material for most emergency department indications, and it is advocated as a first-line test in the pregnant patients [7, 8].

However, US suffers from limits such as operator-dependency, the altered body habitus, a small field of view and the presence of interfering overlying structures, and a negative study may delay diagnosis and therapy; in 30 % of pregnant patients with abdominal pain in whom the US study was negative, subsequent imaging yielded important additional findings, with 64 % of these additional findings requiring surgical intervention [9].

MRI is a versatile, powerful imaging tool that has the potential to give more diagnostic information than any other technique especially in the absence of intravenous (IV) contrast.

CT has contributed to rapid diagnosis and patient triage and has increased emergency department throughput [10]. However the ionising radiation exposure and the potential need for an intravenous contrast material administration imaging technique limit the use of computed tomography (CT) in pregnant patients [9].

The aim of this review is to explain the role of the different imaging techniques for the diagnosis and management of the different causes of acute pelvic pain during pregnancy.

### Imaging tecnique and safety

US is the primary imaging investigation in the diagnostic evaluation of the pregnant patient [11, 12].

Both transabdominal and endovaginal techniques are commonly used to evaluate the uterus, ovaries and other pelvic structures [13].

The disadvantages of ultrasound are its operator dependency and factors such as bowel gas, the gravid uterus and obesity, which may limit the quality of the examination [4].

There are no documented adverse effects on the developing human foetus from diagnostic ultrasound [14]. The US Food and Drug Administration (FDA) proposed an upper limit of 720 mW/cm² for spatial-peak temporal average intensity for obstetric ultrasound [14]. The Doppler technique is not recommended in the first trimester because of the potential harmful effect of the heating of the tissues [15].

A careful risk-benefit analysis is required before performing CT in pregnancy [16, 17].

When CT is used in pregnant patients, it is imperative to use automatic exposure control to reduce the radiation exposure. Protocols should minimise the use of multi-phase studies and should optimise settings to reduce the dose as much as possible without losing image quality. It is common practice to wrap areas adjacent to those being scanned with shielding. Indeed, this may provide a psychological benefit to the patient and her physicians [13].

CT is the investigation of choice when there is a life-threatening situation and a rapid diagnosis is required. The great value of CT is that it can cover many organ systems and large patient volumes rapidly. CT is a primary tool in the case of hypovolemic blunt or penetrating trauma or severe sepsis when a variety of sites of injury or infection need to be evaluated [18].

MRI provides a good overall topographic display and high intrinsic soft-tissue contrast, and also benefits from the lack of ionising radiation [19–21], making its use safe in pregnant patients.

MRI offers different potential advantages such as multiplanar imaging capabilities and the ability to detect and distinguish blood from other fluid collections [19, 22].

A comprehensive multiplanar imaging protocol is used to evaluate the most common causes of abdominal pain. The field of view for the examination extends from the dome of the liver superiorly through the symphysis pubis inferiorly. The protocol includes breath-hold multiplanar T2-weighted sequences based on the half-Fourier reconstruction technique (half-Fourier RARE or single-shot fast spin-echo) and balanced gradient-echo sequences (FIESTA, true FISP), axial and sagittal T1-weighted gradient-recalled echo (GRE) sequences and axial and sagittal diffusion sequences. The time required for this MR protocol is 20 min (Table 1) [18].

**Table 1:** MR protocol for the pelvis during pregnancy

Parameter	Balanced gradient-echo sequence (FIESTA, true FISP, BSSFP)		T2 half-Fourier sequence (HASTE)		T1 3D FS gradient echo sequence DWI	
	Axial	Coronal/sagittal	Axial/axial FS	Coronal/sagittal	Axial/sagittal	Axial/sagittal
Repetition time/echo time (ms)	4.3/2.2	4.3/2.2	1,000/90	1,000/90	4.1/1.1	3,200/75
Flip angle (°)	50	50	150	150	10	10
Field of view (mm)	320–400	320–400	320–400	320–400	320–400	320–400
Matrix	256 × 224	256 × 224	256 × 224	256 × 224	256 × 224	256 × 192
Parallel imaging factor	2	2	2	2	3	2
Section thickness (mm)	5	5	4	4	2.5	10
Intersection gap (mm)	0	0	0	0	0	0
NEX	1	1	1	1	1	6
Receiver bandwidth	125	125	62.50	62.50	62.50	1,930

Diffusion-weighted MR images were acquired with b values of 50, 400 and 800 s/mm²

*FIESTA* Fast imaging employing steady-state acquisition, *FISP* fast imaging with steady-state precession, *BSSFP* balanced steady-state free precession, *HASTE* half-Fourier single-shot turbo spin-echo, *FS* fat saturated

Because of active organogenesis in the first trimester, the absolute safety of MR imaging during this period is difficult to establish.

MR imaging is best avoided unless the potential benefits outweigh the theoretical risks. This statement refers to machines in clinical use at 1.5 T or less. The safety of MR at 3 T has not yet been proven.

The principles guiding the use of MR imaging in pregnancy are to avoid any potential harm even where there are no firm data indicating this has occurred previously.

Therefore examinations should be performed using the minimum thermal and acoustic energy dissipated in the foetus to achieve a clinically useful diagnosis [18]. Because of the known association between gadolinium contrast agents and nephrogenic systemic fibrosis (NSF), concerns have been raised regarding the use of gadolinium in pregnancy [23–26]. Gadolinium-based contrast agents cross the placenta and are excreted by the foetal kidneys into the amniotic fluid [24]. Despite the lack of any evidence of adverse effects after MR studies in the human foetus [25], gadolinium-based contrast agents are classified as category C drugs by the FDA and should only be administered to a pregnant patient “if the potential benefit justifies the potential risk to the foetus and using the smallest dose of the most stable gadolinium agent” [26].

## Obstetric causes

### Early pregnancy failure

Spontaneous abortion occurs in approximately 10–12 % of known first trimester pregnancies [27]. Although the patient may be asymptomatic, spontaneous abortion commonly results in pain and vaginal bleeding.

Ultrasound is the initial diagnostic test of choice for a first trimester patient with pain and bleeding. Correlating sonographic findings with the maternal serum level of β-HCG can help to indicate whether early pregnancy failure has occurred.

Ultrasound can confirm early pregnancy failure with high specificity if no foetal cardiac activity has been detected by the time the embryo measures 5 mm in length or if the pregnancy is known to be 6.5 weeks without an embryo with a heartbeat [28].

When the ultrasound examination either shows worrisome features or is inconclusive, such as in cases with an embryo smaller than 5 mm without a heartbeat, follow-up ultrasound is indicated.

The most widely accepted “discriminatory” sizes of the gestational sac using endovaginal ultrasound are an 8-mm mean sac diameter by which a yolk sac must be visualised and a 16-mm mean sac diameter by which an embryo must be visualised for the pregnancy to be considered normal [29, 30].

Worrisome findings on ultrasound also include slow embryonic cardiac activity, an irregular gestational sac and low position of the gestational sac [31]. Embryonic heartbeat rates below 80 beats per minute (bpm) at 6.0–6.2 weeks or below 100 bpm at 6.3–7.0 weeks’ menstrual age are associated with a very high rate of early pregnancy failure [32].

### Ectopic pregnancy

Ectopic pregnancy is the main cause of pregnancy-related death during the first trimester in the USA with an occurrence of 1:150 births. The most common risk factors are tubal surgery, infections, prior ectopic paregnancy and use of an intrauterine device [IUD]; the symptomatology is characterised by amenorrhoea, abdominal pain, adnexal masses and vaginal bleeding. Ectopic pregnancy is usually tubal (97 %); more rarely , it is ovarian (1 %), interstitial (3 %), abdominal (<1 %) and cervical (<1 %). When ectopic pregnancy involves the intramural portion of the tube, the highest rate of morbidity and mortality is seen [33].

In a woman of reproductive age with symptoms of acute pelvic pain, a serum β-human chorionic gonadotropin (β-hCG) test is usually performed. The correlation between a serum β-hCG level above a discriminatory zone of 1,000 to 2,000 mIU/ml and the absence of a gestational sac in the uterus on transvaginal sonography is highly suspicious for an ectopic pregnancy [34–37].

Sometimes ultrasound may conclusively diagnose ectopic pregnancy if it shows an extrauterine gestational sac with a yolk sac or embryo; more frequently, ultrasound is only suggestive of an ectopic pregnancy showing the presence of an adnexal mass (the most common sonographic finding in ectopic pregnancy) and pelvic free fluid [34, 38, 39]. The adnexal mass usually appears as a sac-like ring, solid or complex. The presence of fluid-containing echoes, correlating with haemoperitoneum, has a 93 % positive predictive value for ectopic pregnancy [40].

In spite of this, ultrasound presents several problems for the differential diagnosis: a corpus luteum might have the same “ring of fire” that characterises the ectopic gestational sac; the finding of an echogenic mass may be due to either an ovarian mass or an ectopic pregnancy and haemoperitoneum can be caused by both ruptured ectopic pregnancy and ruptured haemorrhagic cyst [41].

**Fig. 1:**
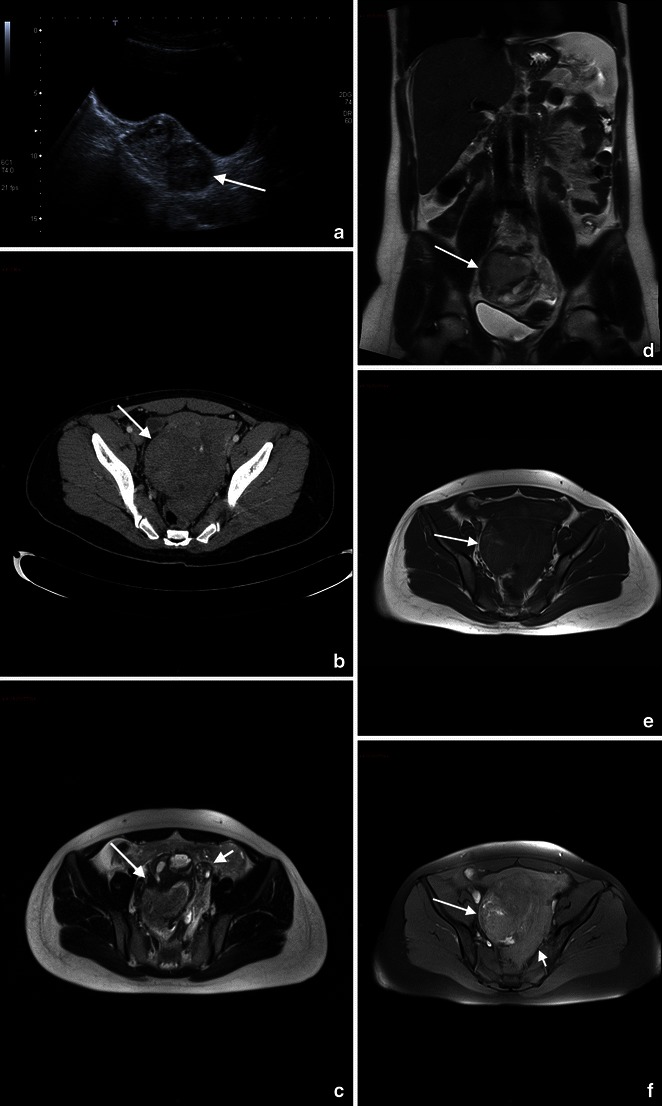
A 27-year-old woman presenting at the emergency department with acute pelvic pain. Abdominal US (**a**) shows a rounded lesion adjacent to the uterus, clearly separate from the left ovary. Axial CT scan shows a voluminous mass in the pelvic cavity (**b**); for the characterisation an MRI was required. Axial (**c**) and coronal (**d**) T2-weighted and T1-weighted images (**e**) of the pelvis show a right heterogeneous adnexal mass (arrow) with fallopian tube haematoma. Note the normal ovary (short arrow) in (**c**). Pre-contrast T1-weighted fat-saturated image (**f**) shows bloody ascites (short arrows). These findings are due to ectopic pregnancy with tubal rupture and haemoperitoneum

**Fig. 2:**
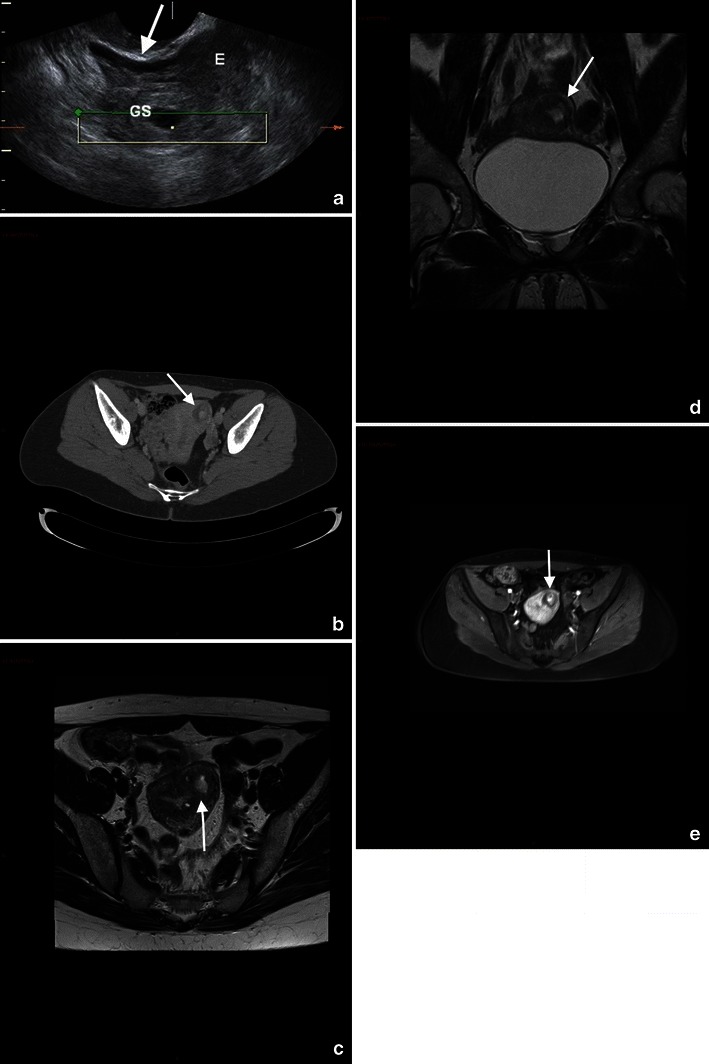
A 35-year-old woman presented with amenorrhea for 7 weeks, abdominal pain and vaginal bleeding; the B-HCG level was elevated. Transvaginal 3D sonogram in sagittal scan shows the interstitial portion of the tube (arrow) located between the gestational sac (GS) and endometrium (**e**). CT of the pelvis (**b**) shows a small lesion on the left side of the uterus. Axial (**c**) and coronal (**d**) T2-weighted images and axial contrast T1-weighted image (**e**) of the pelvis show a left gestational-like structure measuring 10 mm in diameter, surrounded by a thick wall according to interstitial pregnancy

When sonography is indeterminate MRI can be used as a problem-solving technique because of its multiplanar capabilities. MRI can help to diagnose the less common nontubal forms and to differentiate between eccentric implantation in the endometrium and an interstitial ectopic pregnancy (Fig. 1). An interstitial ectopic pregnancy will appear as a gestational sac localised in the cornual aspect of the uterine wall and will be separated from the endometrium by an intact junctional zone [42] (Fig. 2).

Moreover MRI can be useful in differentiating intrauterine pregnancy associated with congenital structural uterine abnormalities from an ectopic interstitial pregnancy, and it can add information about haemorrhagic ascites and haematosalpinx; finally it can characterise adnexal masses and localisation of haematoma [43].

### Placental abruption

Placental abruption often begins with vaginal bleeding and pelvic pain. It is defined as in utero separation of the placenta from the myometrium and causes 10–25 % of prenatal deaths [44, 45].

The three types of placental haematoma are retroplacental, subchorionic and subamniotic. Retroplacental haematomas, posterior to the placenta, represent 43 % of haematomas; subchorionic haematomas, between the chorion and the endometrium, represent approximately 57; subamniotic ones, located between the amnion and chorion, are rare [46–48].

The US diagnostic performance for the diagnosis of abruption is low [49, 50]; in fact 25–50 % of haematomas, mostly retroplacental, remain undetected [51–53] because the echotexture of recent haemorrhage is similar to that of the placenta [49] or because of the small dimensions; moreover clots resulting from chronic abruption may drain through the cervix [53]. The most accurate ultrasound criteria for placenta abruption (sensitivity 80 %, specificity 92 %) are the detection of pre-/retroplacental collections, evidence of marginal subchorionic or intra-amniotic haematomas, increased placental thickness (>5 cm) and jelly-like movements of the chorionic plate [51, 53].

Because of the low sensitivity of sonography in detecting small retroplacental or submembranous haematomas or the occasional absence of bleeding with placental abruption, negative sonographic findings do not rule out the presence of placental abruption [49].

MR imaging is superior to US in the evaluation of placenta haemorrhage because it improves soft tissue contrast and has a wider field of view [54–56].

MR diffusion-weighted imaging (DWI) is an excellent sequence for detecting intrauterine haemorrhagic lesions. Blood breakdown products cause susceptibility effects and can be accurately demonstrated with the diffusion-weighted sequence [57, 58].

The diffusion- and T1-weighted sequences (sensitivity respectively 100 % and 94 %; diagnostic accuracy respectively 100 % and 97 %) are more accurate than the T2-weighted half-Fourier RARE (sensitivity 94 %; diagnostic accuracy 87 %) and true FISP sequences (sensitivity 79 %; diagnostic accuracy 90 %) in the detection of placental abruption [55, 56, 59]. T2-weighted half-Fourier RARE and true FISP sequences have high sensitivity in the detection of acute ischaemic lesions [59 ] and good diagnostic accuracy in the detection of placental haematomas, probably owing to the coexisting condition of acute or subacute bleeding and chronic ischaemia in abruption [47].

**Fig. 3:**
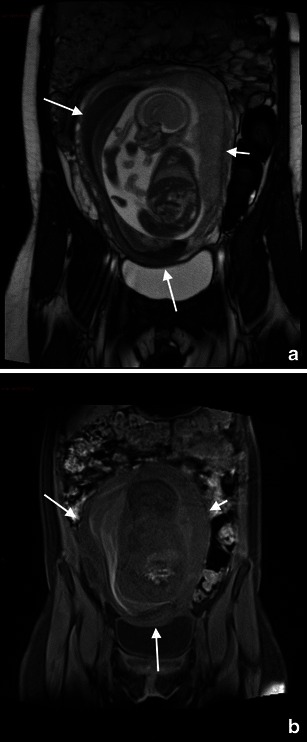
A 25-year-old woman at 28 weeks’ gestation with acute pelvic pain and vaginal bleeding. Coronal T2-weighted image (**a**) shows the intrauterine clot with hypointense areas placed along the right side of the uterine cavity and extended inferiorly to cover the uterine ostium. Coronal T1-weighted fat-saturated gradient-echo image shows the hyperintense subchorionic haematoma (**b**). Note the normal placenta located on the left side (short arrow)

Subchorionic or retroplacental haemorrhage shows low T2-weighted and intermediate to high T1-weighted signal (Fig. 3).

T1- and T2-weighted sequences are both required for complete tissue characterisation. By considering the signal intensity changes with special reference to the paramagnetic effects of methaemoglobin [60], it is possible to estimate the age of the bleeding and to classify intrauterine haematomas as: hyperacute (first few hours, intracellular oxyhaemoglobin), acute (1–3 days, intracellular deoxyhaemoglobin), early subacute (3–7 days, intracellular methaemoglobin), late subacute (≥14 days, extracellular methaemoglobin) and chronic (>4 weeks, intracellular haemosiderin and ferritin). In conclusion MR is very accurate in identifying placental abruptions, even in cases with negative US findings.

In trauma patients who have been subjected to a CT, a systematic evaluation of the placenta excludes the placental abruption with a reported sensitivity of 100 %; however, the specificity is significantly improved with knowledge of the normal placenta and decreases greatly without special training [61].

Placental abruptions were characterised by large, contiguous and retroplacental and/or full-thickness areas of low enhancement that form acute angles with myometrium [61].

Abruptions involving >50 % of the placental surface are frequently associated with foetal demise [46].

### Placental adhesive disorders

Placental adhesive disorders (PAD) include placenta accreta, placenta increta and placenta percreta and are caused by a defect of the decidua basalis that allows the invasion of chorionic villi into the myometrium [58, 62]. Placenta accreta is the least severe form with penetration of the decidua by the chorionic villi, placenta increta is penetration of the myometrium by the chorionic villi and placenta percreta is the most severe one with invasion of both the myometrium and uterine serosa [63]. Prior caesarean section and placenta previa are the two major risk factors [58].

Pelvic US is the most commonly used imaging modality for the diagnosis of PAD [64–66]. Sonographic features include loss of the normal hypoechoic retro-placental myometrium zone, thinning or disruption of the hyperechoic uterine serosa-bladder interface, presence of focal exophytic masses and the presence of lacunae in the placenta (this is the most predictive sonographic sign showing a sensitivity of 79 % and a PPV of 92 %) [66, 67]. Power and colour Doppler can be useful in the diagnosis of placenta accreta because it highlights areas of increased vascularity with dilated blood vessels that cross the placenta and uterine wall [64, 68–70].

**Fig. 4:**
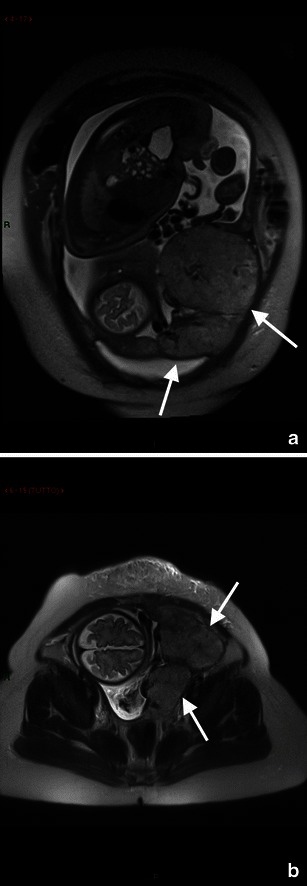
Coronal (**a**) and axial (**b**) T2 HASTE sequences show multiple irregular areas of the placenta bulging into the myometrium with massive invasion of the left parametrium (arrow). These findings are indicative of placenta percreta. An hysterectomy was performed at delivery, which confirmed the presence of placenta percreta

MR findings of more severe disease include dark placental bands on T2-weighted images, with loss of normal low-signal intensity myometrium, disorganised architecture of the adjacent placenta, a focal exophytic mass and, in case of invasion involving the bladder, thinning of the uterine serosal-bladder interface, focal signal in the bladder wall and extension of intermediate signal placental tissue beyond uterine margins with loss of fat planes between the uterus and pelvic organs. [55, 69–72] (Fig. 4).

MRI has potential benefit compared with US because it provides a larger field of view, allowing an easier evaluation of the topography of placental invasion [47].

Many authors recommend a two-stage approach to optimising diagnostic yield, beginning with ultrasound in patients with clinical risk factors and then proceeding to MR imaging for equivocal cases especially in patients with posterior placenta and previous myomectomy [62, 64, 73–75].

Other authors have suggested that MR imaging can better define areas of abnormal placentation, modify levels of invasion, ultimately change surgical management and should be used routinely [55, 73].

## Ginecologic causes

### Uterine rupture

Uterine rupture is a rare, catastrophic event that often presents with severe abdominal pain. Predisposing factors include previous uterine surgery, including caesarean deliveries and myomectomy, and congenital uterine malformations [76].

When uterine rupture occurs intrapartum, abdominopelvic ultrasound shows a bulky empty uterus with an anterior hypo-/anechogenic line corresponding to the uterine tear, the foetus and placenta in the abdominal cavity and increased intraperitoneal fluid [77, 78].

**Fig. 5:**
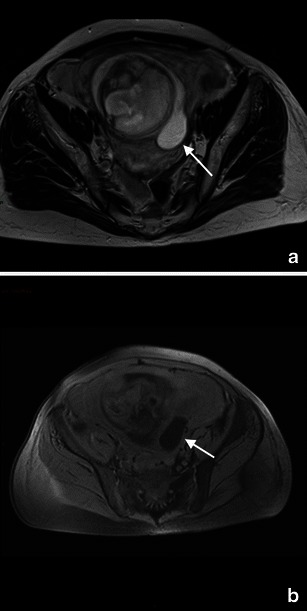
A 38-year-old woman was admitted at 26 weeks’ gestation presenting with vomiting and acute abdominal pain. Axial T2-weighted HASTE (**a**) and T1-weighted fat-saturated sequences (**b**) show posterior extravasation of amniotic fluid into a hernial sac that contains a small fluid level; these findings are suggestive of a sealed uterine rupture. Note the presence of haemoperitoneum

MRI allows clear visualisation of the uterine wall; therefore, it helps to diagnose both ante-partum uterine rupture in patients with indeterminate ultrasound evidence, showing the tear itself [79] and other uterine wall defects including uterine dehiscence (separation of the myometrium with preservation of the overlying peritoneum and internal foetal membranes) [80], and uterine sacculation (uterine wall ballooning because of a functional weakening of the myometrium) [81] (Fig. 5).

### Adnexal masses

Adnexal masses occur in approximately 2 % of all pregnancies [3]. Adnexal masses are not a usual cause of pain, with 65 % of these masses being asymptomatic and discovered incidentally on physical examination or sonography [82].

The most common ovarian mass encountered in pregnancy is a benign ovarian cyst [3]. There are many types of benign ovarian cysts including corpus luteal, follicular, haemorrhagic and endometriotic. An adnexal mass can be complicated by torsion, haemorrhage or rupture and in this cases may present with pain [44].

Most masses can be accurately assessed by ultrasound [83, 84]. However, MR imaging can provide further characterisation, particularly for evaluating their haemorrhagic content, evident as high signal intensity on T1-weighted sequences with no signal loss on fat suppression. It can identify an exophytic/pedunculated leiomyoma by showing its stalk, a band of tissue with associated bridging vessels connecting the mass to the uterus [85].

**Fig. 6:**
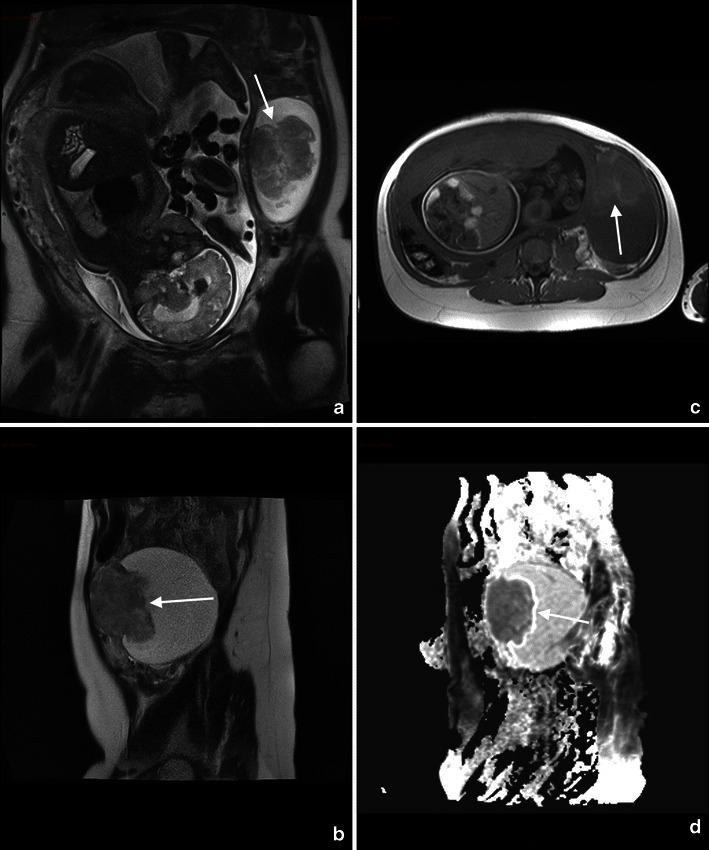
Coronal (**a**) and sagittal (**b**) T2-weighted MR images show a complex mass, with fluid and solid components on the left ovary. Haemorrhagic areas are seen on T1-weighted sequence (**c**). Sagittal DWI image shows reduction of diffusion in relationship to the high cellularity of the solid component of the mass (**d**). Cystoadenocarcinoma was confirmed at surgery

Functional ovarian cysts can be distinguished from ovarian neoplasms at MR imaging because of the presence of papillary projections and nodular septa in neoplasms [86]. The most common ovarian neoplasm found in pregnancy is the benign cystic teratoma, which arises from ovarian germ cells. At MR imaging, these lesions have high signal intensity on T1-weighted images and intermediate signal intensity on T2-weighted images owing to the high-lipid-content cyst fluid. The fat in these lesions can be further verified on MR images by using frequency-selective fat saturation (Fig. 6).

### Ovarian torsion

Ovarian torsion is increased during pregnancy and complicates 1 in 800 pregnancies. Torsion can also occur in a normal ovary, usually the right one. Ovarian torsion most often occurs between 6 and 14 weeks’ gestation when uterine enlargement is most rapid [87, 88].

The pelvic transvaginal US represents the first step in the diagnosis: initially it is possible to observe an increase in the ovarian volume, with displaced follicles on the edge. Hyperechoic areas, signs of bleeding infarction associated with hypoechoic areas and expression of interstitial oedema may be present in the ovary. In 94 % of cases, the absence of venous flow has an elevated value predicative of ovarian torsion; the opinion on the role of pulsed Doppler or colour Doppler is discordant; although in the presence of an enlarged ovary, oedematous and painful, the absence of flow is highly suggestive of adnexal torsions [89].

When US diagnosis is difficult, MRI can be used. MRI findings include an oedematous and thick vascular pedicle with haemorrhagic signal intensities within the ovary [13].

It is recommended to perform a T1- and fat-suppressed T1-weighted sequence to detect haemorrhage [90]. Contrast-enhanced, fat-suppressed, T1-weighted images can be used to detect the absence of vascular supply.

**Fig. 7:**
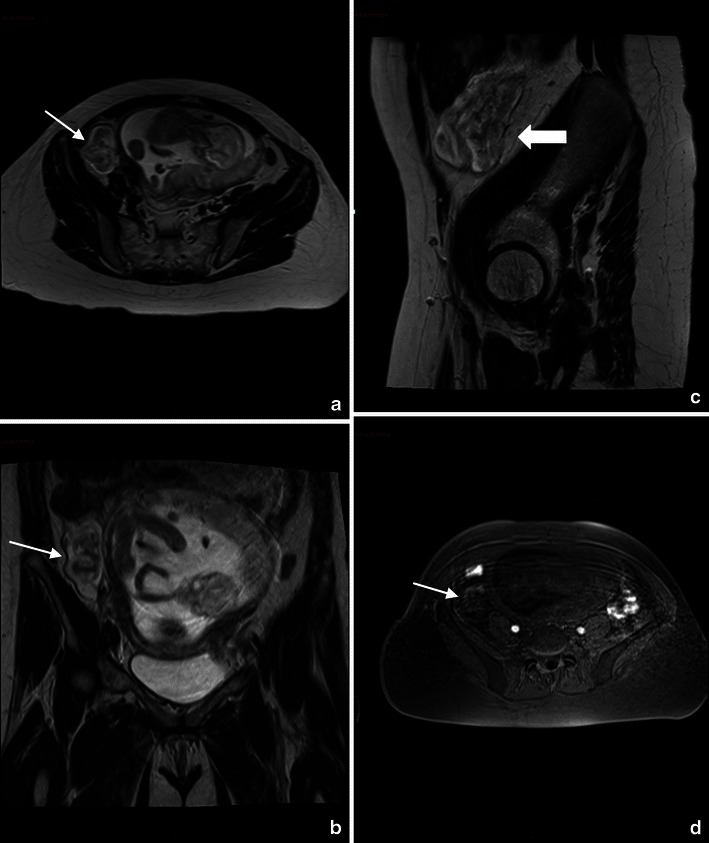
Ovarian torsion in a 38-year-old woman at gestation week 26 with acute pelvic pain. Axial (**a**), coronal (**b**) and sagittal (**c**) T2-weighted sequences show an enlarged, oedematous right ovary (arrow). Axial T1-weighted VIBE fat-saturated sequence (**d**) shows areas of hypersignal in the context of the right ovary indicative of haemorrhagic infarction

MR imaging features of ovarian torsion include an enlarged ovary and a thickened, twisted fallopian tube [91]. On T1-weighted images, the signal intensity varies according to the age of internal blood products. Late torsion demonstrates increased signal intensity on T2-weighted images owing to necrosis [92] (Fig. 7).

The oedema, determined by the impeded venous outflow, which results in the increase of the ovarian dimensions, is manifested by an increased signal in the T2-weighted sequence.

Sagittal MR imaging may be helpful in detecting a thickened tube, which appears as a tubular protrusion on the twisted side [90].

Although it is preferable to use US and MR in the diagnosis of ovarian torsion, it can be necessary to do a CT under emergency conditions, for example, in the presence of a massive haemoperitoneum.

### Leiomyoma

Fibroids (leiomyoma) are the most common pelvic tumours affecting females in the fertile age group. One in 500 pregnant women experience acute abdominal pain with uterine tenderness and possibly low-grade fever owing to leiomyoma-related complications, mostly the result of haemorrhagic infarction [93, 94].

Approximately half of all leiomyomas grow during pregnancy, mainly in the first trimester because of rising oestrogen levels [95]. Abdominal pain and uterine contractions can result from necrosis and degeneration of leiomyomas secondary to rapid growth.

“Red degeneration” is the most common type of degeneration during pregnancy and occurs when a leiomyoma outgrows its blood supply with resulting haemorrhage. Such leiomyomas can appear on ultrasound as circumscribed masses with cystic spaces or heterogeneous.

Ultrasound features in acute haemorrhagic infarction (red degeneration) include heterogeneous or hyperechoic lesions. Later, leiomyomas may have anechoic components resulting from cystic necrosis, which allows confirmation of the diagnosis [96, 97].

MRI can be helpful in making the diagnosis.

Leiomyoma undergoing haemorrhagic degeneration during pregnancy typically exhibit diffuse or peripheral high signal intensity on T1-weighted imaging and variable signal intensity on T2-weighted imaging [98].

The hyperintense rim on T1-weighted imaging may correspond to obstructed veins at the periphery of the mass.

### Nephrolithiasis

Kidney stones (nephrolithiasis) are not very common during pregnancy. The incidence of the symptomatic cases is estimated to be up to 1 in 2,000 pregnancies [99–103], and it is similar between pregnant and non-pregnant women. Renal colic is one of the most frequent non-obstetric causes for abdominal pain and subsequent hospitalisation during pregnancy [104, 105].

Nephrolithiasis typically manifests in the second or third trimester with equal involvement of the right and left sides [17, 82].

In pregnancy anatomical changes include dilatation of the renal calyces, pelvis and ureters due to the compression of the pregnant uterus and the effect of progesterone on the ureteral smooth muscle [106]. The physiological changes include increased renal plasma flow and glomerular filtration rate [107], causing a state of hypercalciuria and hyperuricosuria [104].

Ultrasonography (US) is usually the primary imaging modality for evaluation of hydronephrosis and urolithiasis during pregnancy.

Ultrasound is the first imaging test for suspected urolithiasis in pregnancy, despite its substantial limitations and a reported sensitivity as low as 34 % [108, 109].

False negatives are rare and due to obstruction without dilatation, but false positives are common because of the dilatation of the collecting system that occurs physiologically in pregnancy.

Ultrasound can identify stones within the renal pelvis but direct demonstration of ureteral calculi is difficult owing to the gravid uterus. Stones at the ureterovesical junction may be detected using transvaginal ultrasound. Doppler techniques have been evaluated as an adjunct [109, 110].

Colour Doppler may show the presence of the twinkling artefact at the level of the stone even at sites where differentiation of the hyperechoic stone from surrounding hyperechoic tissues may be difficult [111]. Comparison between sides of the resistive index (RI) from intrarenal Doppler waveforms can be helpful in patients with acute obstruction showing a difference of at least 0.04 in RI of intrarenal arteries between the symptomatic kidney and the contralateral one [108].

Colour Doppler can also be used to detect the passage of urine at the ureterovesical junction: the so-called ureteral jet. In the nonpregnant abdomen, absence of this sign on the symptomatic side has a very high sensitivity and specificity for obstruction [109].

However, its diagnostic value is hampered as ureteral jets may be absent in 15 % of asymptomatic pregnant women.

Possible false-positive results can be decreased by imaging patients in the contralateral decubitus position; this manoeuvre reduces the degree of physiological dilatation.

Additional imaging by MR, noncontrast low-dose CT or intravenous (IV) pyelogram may be required if US is negative.

MR urography should be considered as a second-line test when use of US fails to establish a diagnosis and when there are continued symptoms despite conservative management [89].

MR imaging has high sensitivity for detection of urinary tract dilatation and identification of the site of obstruction.

Although MRI does not visualise ureteral calculi, many salient features may suggest the presence of obstructing calculi. Stones appear as signal voids overlying the high signal of urine within a dilated ureter [112].

The presence of a standing column of urine below the level of the pelvic brim, in addition to proximal ureteral dilation, is suggestive of an obstructing distal ureteral calculus (“double kink sign”) [112]. Other MRI features that suggest pathologic rather than physiologic hydronephrosis include an “unusual” site of obstruction (such as the pelvoureteral junction or vesicoureteral junction), an abrupt ending of the ureter (rather than a smooth taper at the level of the pelvic brim), and perinephric or periureteral oedema. In contrast, physiologic hydronephrosis at MRI is characterised by gradual, smooth tapering of the mid to distal ureter due to extrinsic compression between the gravid uterus and iliopsoas muscle. The main limitation of the MR urography is that resolution tends to be less than optimal, and small stones can be missed.

**Fig. 8:**
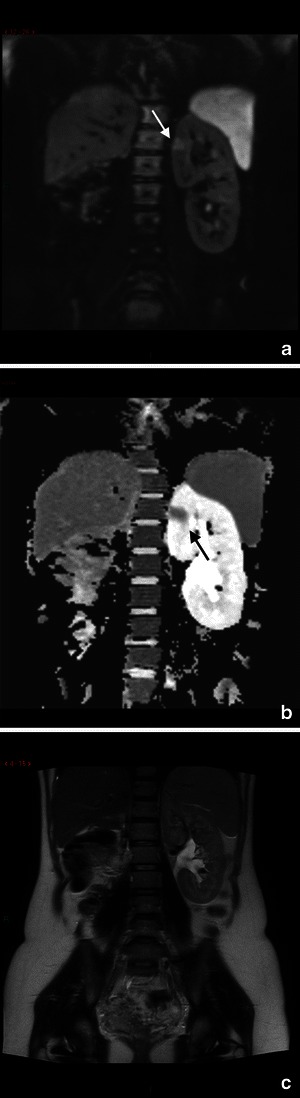
A 33-year-old woman at 32-gestation week was admitted manifesting fever and acute pelvic pain. Coronal diffusion-weighted (DW) image (**a**) and ADC map (**b**) show a focal area of restricted diffusion at the level of the upper pole of the left kidney, not seen at T2 Haste (**c**). The findings are indicative of focal pyelonephritis

MRI is helpful in demonstrating complications such as pyelonephritis that are visualised as an enlarged oedematous kidney [113]. Areas of focal pyelonephritis have lower signal intensity on T2-weighted and restricted proton diffusion on the DW images [114] (Fig. 8).

In unresolved cases, CT remains a reliable technique for depicting obstructing urinary tract calculi in pregnant women.

The average estimated foetal dose, using a low-dose CT technique, was 7 mGy, i.e. below the 50-mGy limit above which there is a statistically higher risk of teratogenesis [17, 115, 116].

## Other causes

### Appendicitis

Appendicitis occurs in about 1 in 1,500 pregnancies and is a difficult diagnosis in pregnancy owing to variable appendiceal position and difficulty with clinical examination of the gravid abdomen [2, 115, 117, 118].

Anatomic and physiologic changes that may disguise and delay the diagnosis of acute appendicitis in pregnant women include a cephalad displacement of the appendix from the right lower quadrant by the enlarged uterus, an increased leukocyte count and a physiologic increase in maternal blood volume that diminishes the ability to recognise tachycardia or hypotension.

Ultrasound is the technique of choice for investigating suspected appendicitis, using the same parameter set for non-pregnant patients, including visualisation of a blind-ending, dilated (>6–7 mm in diameter) aperistaltic and noncompressible tubular structure arising from the caecum [119, 120].

Therefore, if the result of the US is negative or doubtful, without an alternative diagnosis, other imaging techniques are necessary to diagnose or exclude appendicitis.

MR is the method to perform in cases in which the MR scan is collocated in the ED or is however available in a short time.

In suspected appendicitis in pregnancy, intravenous gadolinium is not used.

MR imaging features of a normal appendix include a diameter less than 6 mm, an appendiceal wall thickness less than 2 mm, low luminal signal intensity on T1- and T2-weighted images, and no periappendiceal fat stranding or fluid [92].

**Fig. 9:**
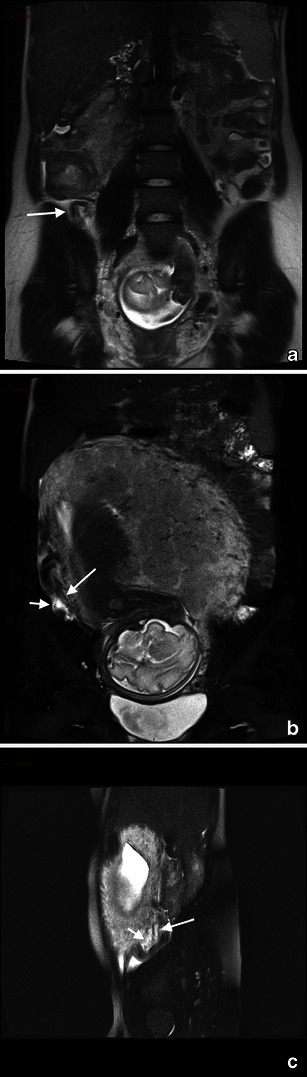
Acute appendicitis in a 27-year-old woman at 34-week gestation presenting with abdominal pain on the right side. Coronal (**a**) T2 image shows a thickened fluid-filled appendix (arrow). Coronal and sagittal T2–weighted HASTE fat-saturated images (**b**, **c**) with high signal intensity of periappendicular fat due to inflammatory changes. At surgery and pathology, the diagnosis of appendicitis was confirmed

MR imaging features of appendicitis include an appendiceal diameter greater than 7 mm, an appendiceal wall thickness greater than 2 mm, appendicoliths and surrounding hyperechoic inflamed fat or hypoechoic fluid on T2-weighted images [120] (Fig. 9).

If MR imaging cannot be performed because of absolute contraindications or is not available, CT is an alternative. The risks of misdiagnosis without accurate imaging outweigh the small potential risk of ionising radiation.

### Bowel obstruction

In the gravid patient, ultrasound is the first choice in the evaluation of bowel conditions other than appendicitis. Bowel obstruction in pregnancy is fairly uncommon (1 per 2,500 to 1 per 3,500 pregnancies). It is usually due to adhesions (60–70 %), less commonly due to volvulus (≈25 %) [117]. In long-standing or high-grade obstruction, ultrasound may show dilated loops of bowel with fluid levels and aperistalsis, but depiction of the point or cause of bowel obstruction usually remains undetermined. Magnetic resonance studies for bowel obstruction, performed with the use of multiplanar T2-weighted singleshot fast spin-echo (SSFSE) imaging, do not have extensive validation, but can accurately depict the site of small bowel obstruction in approximately 70 % of cases [121, 122].

### Infectious diseases

Osteomyelitis in pregnancy is rare and represents a serious threat to the mother and especially to the good outcome of the foetus.

Depending upon the location and degree of disease the patient presents with pubic or back pain, low-grade fever and altered gait, the inflammatory markers are altered in the laboratory tests [123].

The gold standard for diagnosis is represented by tissue culture and histopathological examination; it is also possible to identify the specific pathogen responsible and set an appropriate treatment.

MRI is currently considered to have the highest sensitivity and specificity of imaging modalities for detecting acute haematogenous osteomyelitis and is able to identify soft-tissue/joint complications.

On MRI, an alteration of the normal marrow signal intensity is valuable; the oedema and exudates appear as defined low-signal intensity areas on the T1-weighted images and a high signal on T2-weighted and STIR images with diffusion restriction [123].

**Fig. 10:**
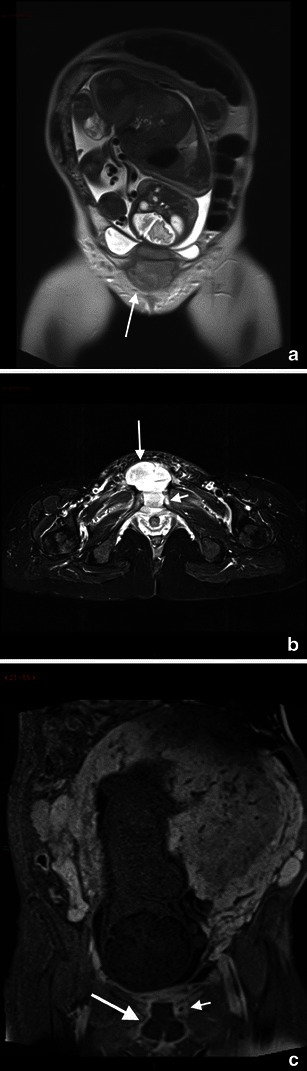
A 35-year-old woman presents with pubic pain, fever and elevated inflammatory markers. Coronal T2–weighted HASTE image (**a**) and T2–weighted STIR image (**b**) show fluid collection (arrow) at the level of the symphisis pubic bone with hyperintensity of the pubic bone (short arrow). Contrast T1-weighted image shows the presence of a thick vascularised wall indicative of an abscess with a small abscess at the level of left pubic bone (short arrow). These findings are in agreement with osteomyelitis

On MRI, a sequestrum is seen as a low signal intensity structure on T1-weighted and STIR sequences, whereas the surrounding granulation tissue has intermediate to low signal intensity on T1-weighted images and high signal intensity with STIR or T2-weighted sequences [116] (Fig. 10).

## Conclusion

Determining the cause of acute pelvic pain in pregnant women can be difficult because of the multiple confounding factors found in normal pregnancy.

Pelvic ultrasound is the preferred primary imaging investigation but it may be of limited value because of the altered body habitus, small field of view and presence of interfering overlying structures. MR imaging is extremely accurate in identifying both obstetric and non-obstetric causes and should be used when ultrasound findings are non-diagnostic or equivocal.

In the unresolved cases, CT remains a reliable technique for depicting obstructing urinary tract calculi in pregnant women.
